# Under-utilisation of reproducible, child appropriate or patient reported outcome measures in childhood uveitis interventional research

**DOI:** 10.1186/s13023-019-1108-3

**Published:** 2019-06-06

**Authors:** Ameenat L. Solebo, Robert J. Barry, Pearse A. Keane, Jugnoo S. Rahi, Alastair K. Denniston

**Affiliations:** 1 NIHR Moorfields Eye Hospital Biomedical Research Centre, London, UK; 2 NIHR Great Ormond Street Hospital Biomedical Research Centre, London, UK; 3 University College London Great Ormond Street Institute of Child Health, London, UK; 4 University College London Institute of Ophthalmology, London, UK; 5 Institute of Translational Medicine, Birmingham Health Partners, University of Birmingham, Birmingham, UK; 6 Department of Ophthalmology, Queen Elizabeth Hospital Birmingham, University Hospitals Birmingham NHS Foundation Trust, Birmingham, UK; 7 Moorfields Eye Hospital NHS Foundation Trust, London, UK; 8 Clinical and Academic Department of Ophthalmology, Great Ormond Street Hospital NHS Foundation Trust, London, UK; 9 Institute of Translational Medicine, Birmingham Health Partners, Queen Elizabeth Hospital Birmingham, Edgbaston, Birmingham, B15 2WB UK

**Keywords:** Paediatric uveitis, Rare disease, Outcome measures

## Abstract

**Background:**

Childhood uveitis is a collection of chronic rare inflammatory eye disorders which result in visual loss in at least one eye of one fifth of affected children. Despite the introduction of novel systemic immunochemotherapies, it remains a blinding disease.

We have undertaken a systematic review of outcome measures used in interventional trials of children with, or at risk of uveitis, in order to investigate metric quality and heterogeneity, as possible barriers to the translation of clinical research into improved outcomes.

**Methods:**

Systematic review of trials registered within databases approved by the International Committee of Medical Journal Editors (ICMJE). Eligible trials for were those which involved participants aged under 18 years with or at risk of non-infectious uveitis. Data on date of study commencement, uveitis site, inclusion age criteria, and outcome measure characteristics including type, dimension and quality were extracted independently by two authors. Quality was determined using the reproducibility, validity and age-appropriateness of the metric.

**Results:**

Of 917 identified trials, 57 were eligible for inclusion. Twenty different domains across five dimensions were used as primary outcome measures. The structure most commonly used was multiple separate outcome measures. In a quarter of studies, outcomes were assessed less than 3 months following the intervention. Disease activity was the most commonly assessed dimension, with only 30 studies (60%) using reproducible methodologies to assess activity. Only 2/12 (18%) studies on intermediate or posterior uveitis used reproducible activity grading schemes. Of 18 studies involving children aged under 6 years old which used outcome measures related to visual function, only 8/18 (44%) described the use of age-appropriate acuity assessment measures. None of the studies used a vision related quality of life metrics which had been validated for use in childhood.

**Conclusion:**

This review of outcome measures in childhood uveitis interventional trials has identified under-utilisation of reproducible or child appropriate measures, and considerable heterogeneity in metric type, and structure. Clinicians and researchers interested in improving outcomes for affected children must identify a patient and family centred core outcome set, and work to validate both objective and patient (or proxy) reported disease age appropriate outcome measures.

**Electronic supplementary material:**

The online version of this article (10.1186/s13023-019-1108-3) contains supplementary material, which is available to authorized users.

## Background

Uveitis is a descriptive term used for a group of rare inflammatory eye diseases which can result in structural ocular abnormalities and visual disability. Childhood uveitis, with an estimated incidence of 5/100,000 children per annum, accounts for less than 5% of all uveitis cases [1–3]. Almost one in five affected children lose vision in at least one eye by the time they reach adulthood [4–6]. Uveitis differs in children and adults in aetiology, natural history, management and response to therapeutics [3]. ,Childhood uveitis is particularly challenging due to the heterogeneity of clinical presentation, difficulties in diagnosing affected children, and the delicate balance between the risk of insufficient control of inflammation with the potential negative impact of immunosuppressive chemotherapy during critical periods of ocular and general development [7]. Although commonly an isolated ocular disorder, uveitis can also occur as a manifestation of a multisystem inflammatory disease [4, 7]. In terms of prevalence, the most important associated disease is juvenile idiopathic arthritis (JIA), which is diagnosed in almost half of all childhood uveitis [7–9]. Rather than being a diagnosis per se, JIA is itself an ‘umbrella’ term for a group of idiopathic multisystem disorders which result in chronic inflammatory arthropathy [10].

Uveitis is classified anatomically, affecting anterior, intermediate and / or posterior ocular structures, through clinical assessment by an ophthalmologist using slit lamp biomicroscopy [11]. In 2005, a multinational collaborative group developed the Standardization of Uveitis Nomenclature (SUN) [11], which included ordinal scales for disease activity quantification using this approach (Table 1). Prior to this points several different non-coterminous ordinal scales were in use internationally.^10^ Although vision is the key outcome of interest for any ocular disorder, the SUN scales were adopted as outcome measures for disease monitoring in clinical practice, and as surrogate endpoints for the irreversible, blinding damage caused by chronic intraocular inflammation [13]. Other potential outcomes of interest for childhood uveitis, as identified through consensus work by the Multinational Interdisciplinary Working Group for Uveitis in Childhood (MIGWUC) [12], include the incidence of sight threatening structural ocular complications, and the disease related impact on the child’s visual and global function, and on quality of life. As yet, there is no uveitis specific quality of life instrument validated for use in children, and two generic instruments (the Child Health Assessment and the Child Health Questionnaires) are erroneously described by MIGWUC as quality of life metrics rather than functional assessments (Table 1) [12].

**Table 1:** Standardised Uveitis Nomenclature (SUN) Disease activity grading schemes and Multinational Interdisciplinary Working Group for Uveitis in Childhood (MIGWUC) outcome parameters [11, 12]

SUN Grades (2005)	
Anterior chamber cells	Non-linear ordinal scale: 0 (no cells in examined field), 0.5+ (1–5 cells), 1+ (6–15), 2+ (16–25), 3+ (26–50), 4+ (> 50 cells in examined field)
Anterior chamber flare	Non-linear ordinal scale from 0 (no clouding of view of anterior structures) to 4+ (intense fibrin deposition in anterior chamber)
Vitreous haze	Non-linear ordinal scale from 0 (no clouding of view through vitreous) to 4 (unable to see through vitreous gel)
MIGWUC outcome domains (2012)	
Grade of activity in anterior chamber	Slit lamp exam (subjective measure of cells and flare) and laser photometry (objective measure of flare), and number of visits with active uveitis
Visual acuity	Appropriate for age at testing
Development of structural complications	Slit lamp examination for anterior structural complications Presence of high eye pressure or glaucoma (diagnostic protocol not clear) Slit lamp examination or imaging for posterior structural complications
Quality of life	*Child Health Assessment Questionnaire* ^*a*^ *; Child Health Questionnaire* ^*a*^ Pediatric Quality of life Inventory; Uveitis-specific quality of life instrument^b^
Overall uveitis related disability	Visual analog scale scoring undertaken by child, family, ophthalmologist or rheumatologist
Social outcome	School absence
Anti-inflammatory medication	Reduction in corticosteroid use
Surgery	Yes / no
Biomarkers	Research tools

^a^CHAQ and CHQ are functional assessments rather than quality of life metrics

^b^As yet, there is no validated instrument

The advent of systemic immunomodulators has improved the outlook for some affected children [13]. Despite these advances, paediatric uveitis remains a blinding disease [7]. One potential obstacle to improving outcomes for affected children is the quality and heterogeneity of disease outcome measures used in studies which assess the effectiveness of new interventions, and heterogeneity of utilised measures [14]. Interventional trials are “only as credible as their outcomes” [15], and the selection of patient-oriented outcomes is key to the assessment of the efficacy of one intervention over another. Once outcome domains are identified as meaningful and appropriate, the measure used to quantify or qualify the outcome must be able to reliably and reproducibly capture a significant change in disease or health status. Outcome measure heterogeneity is a feature of both uveitis research [14] and paediatric interventional research more broadly [16, 17] and has considerable negative impact on translational research [18, 19], by acting as a barrier to the synthesis and meta-analysis needed for the generation of an evidence base to support clinical practice.

The International Committee of Medical Journal Editors (ICMJE) has developed a system of endorsement for clinical trial databases which meet certain quality requirements. In order to receive ICMJE endorsement, a trial registry must be open access; open to all prospective registrants; managed by a not-for-profit organization; able to continuously ensure the validity of registered data; electronically searchable; and must include the minimum 20-item trial registration dataset before enrolment of the first participant (full criteria available at www.who.int/ictrp/network/trds/en/index.html). Studies must also pre-identify primary outcome measures. These registries form a repository of protocol data for completed and underway studies.

We have undertaken a systematic review of outcome measures used in interventional trials of children with, or at risk of uveitis, in order to investigate metric quality and heterogeneity.

## Main text

### Methods

#### Identification of clinical trial registries

All clinical trial databases that were registered with, and approved by, the ICMJE as of 10th March 2017 were identified [http://www.icmje.org/recommendations/browse/publishing-and-editorial-issues/clinical-trial-registration.html]. All identified national registries were searched and all registries were included regardless of language. (Additional file 1).

#### Review inclusion criteria

Trials eligible for inclusion were those which involved participants aged under 18 years with uveitis, or identified as at risk of uveitis due to juvenile idiopathic arthritis, studies with an interventional methodology, and with outcome measures which related to uveitis (visual acuity, visual function, disease activity, ocular sequelae of uveitis, or vision related quality of life measures). Studies also had to meet the ICMJE criteria for inclusion (Additional file 1).

#### Selection of trials for review

Electronic searches of the trial databases were undertaken using the terms ‘uveitis’ and again using ‘juvenile idiopathic arthritis’ (Additional file 1). Terms were entered either free text or through key word selection, as appropriate. Screening of identified trials was undertaken independently by two investigators (ALS, RB). Where no consensus was reached disagreements were resolved by the senior author (AKD).

#### Data extraction

Data were independently extracted from registered protocols by two authors (ALS, RB), and comprised uveitis site (anterior, intermediate, posterior, panuveitis or mixed), study inclusion age criteria, date of trial registration and study commencement, trial status (active, completed, results available), the time from intervention at which outcome was assessed, and whether the uveitis related outcome was a primary or secondary outcome measure for the study. Data items collected on the characteristics of the primary outcome measures were outcome structure, type, and dimension.

Where trial methodology details were unavailable via the register entry, further information was sought through email contact with the principal investigators for the study. Second emails were sent 4 weeks later, in cases of non-response, with a request for further information. A supplemental search of the literature (Additional file 1) was undertaken in order to identify eligible trials, and to identify any methodology details unavailable in the registered protocols.

#### Analyses

The domain examined within each dimension and quality of the outcome measure was categorised using the panel agreed by the Multinational Interdisciplinary Working Group for Uveitis in Childhood in 2012 (Table 1) [12]. Visual function metrics were categorised as acuity or other function, and determined to be of good quality if the assessment methodology was age appropriate and validated for use. Within the dimension of disease activity, the domain (ie the specific study variable collected for the outcome measure) was described. For the purpose of this review, the metric was determined to be of good quality if reproducible. Reproducibility was defined using the guidance within the MIGWUC consensus panel and SUN guidance. Macular oedema was classified as a marker of disease activity. Structural or functional sequelae of inflammation were defined as a diagnosis of cataract, glaucoma, band keratopathy, hypotony or epiretinal membrane. As no validated objective metric exists for these outcomes, measures dependent on these events were determined to be of good quality if a reproducible measure of clinical pathology was used (eg a published grading scale used to determine severity of cataract) [20]. Patient reported metrics were determined to be of good quality for the purposes of this study if they were validated for use within the target study population, for example the Children and Young Person Vision Related Quality of Life instrument (CYP_VQol) [21], or the Child Health Assessment Questionnaire (CHAQ) [22].

We also performed subgroup analyses of registered interventional trials and outcome measures used by (a) date of registration (before versus after 2005, publication of the Standardisation of Uveitis Nomenclature guidance [11], and 2012, the publication date of the Multinational Interdisciplinary Working Group for Uveitis in Childhood’s Proposed Outcome Measures for Prospective Clinical Trials [12]) and by (b) publication status (results published within peer reviewed literature versus not published) using non parametrical tests (χ2, Mann Whitney U, Poisson). 95% confidence intervals are reported where appropriate. Statistical analyses were performed using StataSE 15 (Stata Corp, Chicago Illinois).

## Results

### Identification of interventional trials relevant to paediatric uveitis

**Fig. 1:**
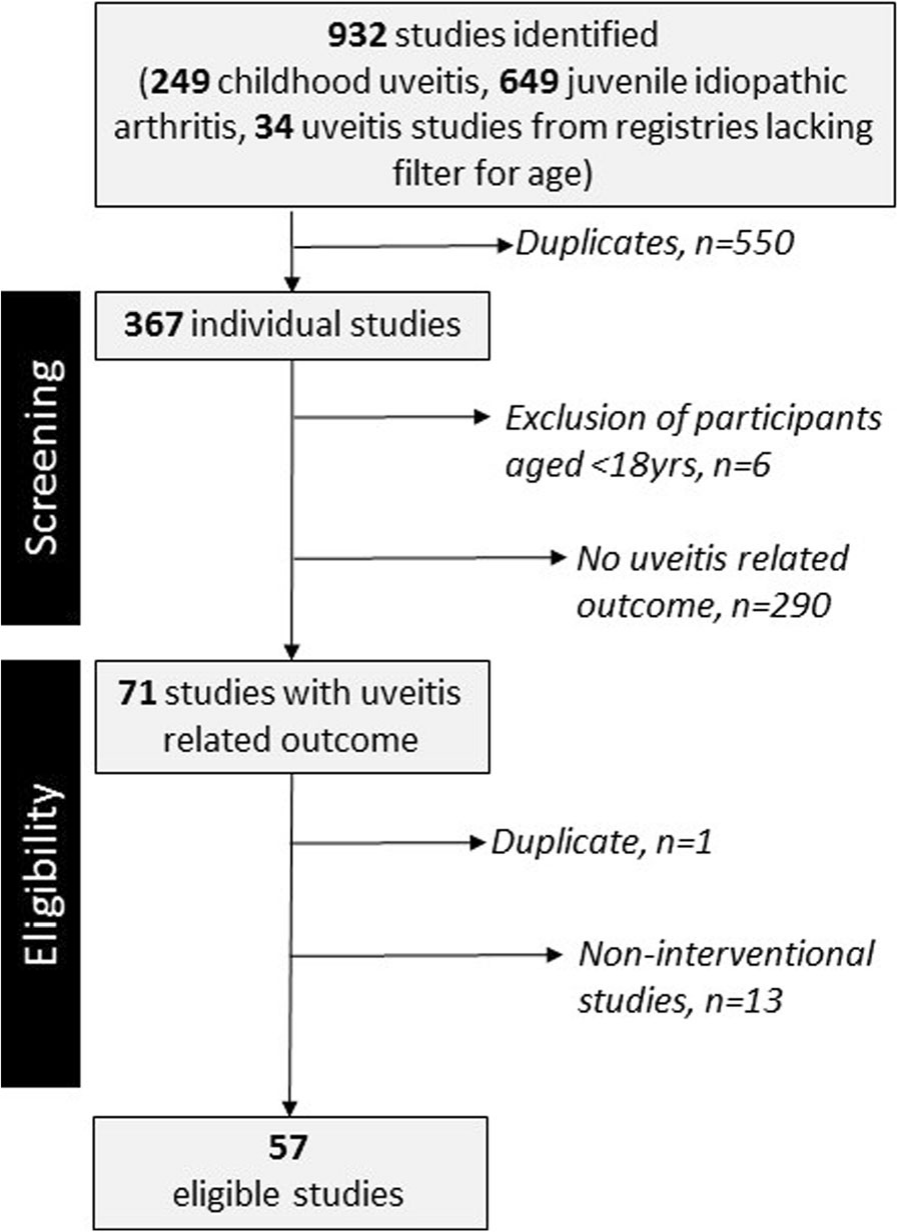
Flow chart depicting the process of identification, screening, and inclusion of uveitis clinical trials for this systematic review

At the time of the database search (10th March 2017), 16 ICMJE endorsed registries were identified, consisting of four international and 12 national registries (Table 2). Searching of these registries identified 917 trials: 249 trials related to childhood uveitis, 649 related to juvenile idiopathic arthritis (JIA), and 34 related to uveitis which were identified through the German and Indian Clinical Trials Register, the only registries for which it was not possible to limit the search to trials involving children. Following removal of duplicate studies, 367 were screened for eligibility, of which 296 were ineligible. Of the 290 studied which were ineligible through the absence of a uveitis related outcome measure (Fig. 1), 184 studies involved investigations of children with JIA. We identified 57 eligible interventional studies involving participants aged under 18 years old. Of these trials, 56 involved a pharmacological agent, and seven of the 56 involved intraocular delivery of the agent. The remaining 6 excluded studies were ineligible due to non-involvement of children aged under 18 years.

**Table 2:** Data extracted from eligible trial protocols

Primary outcome characteristic	Details
Outcome structure	*Single, composite or multiple separate single measures*
Outcome type	*Safety, efficacy or both*
Dimension	*Visual function*
	*Disease activity*
	*Use of anti-inflammatory medication*
	*Structural or functional sequelae of inflammation*
	*Vision related quality of life*
	*Other patient reported outcome measure*

### Characteristics of included trials

**Fig. 2:**
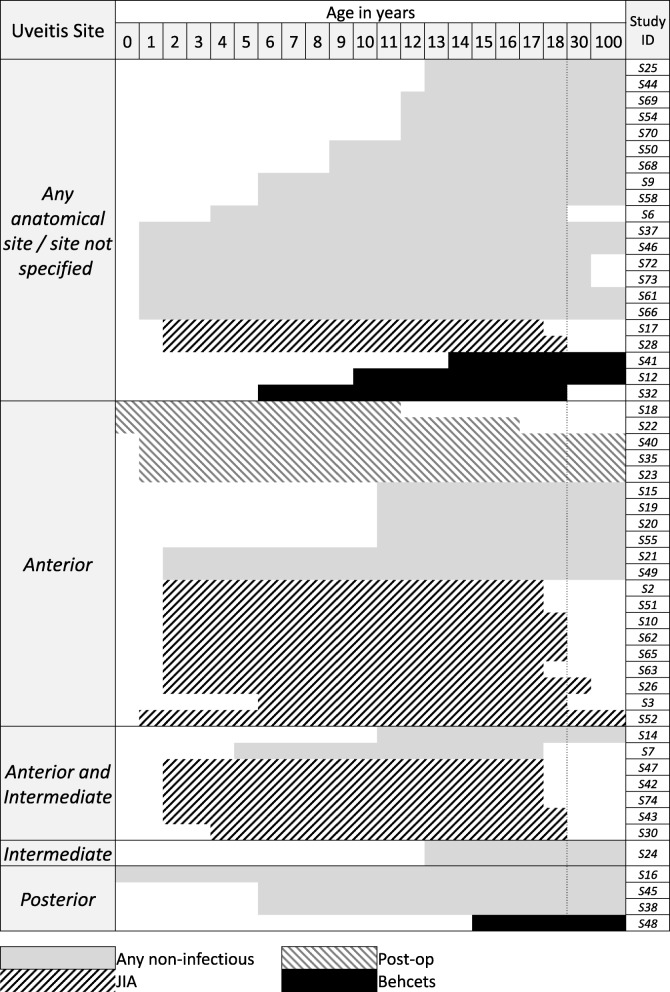
Inclusion age range and disease site for included studies

We identified 31 studies which involved participants with a broad diagnosis of non-infectious uveitis, whilst the remaining studies had specific populations, comprising Juvenile Idiopathic Arthritis (*n* = 16 studies), anterior uveitis secondary to cataract surgery (*n* = 4), Behcets (*n* = 4) and Chronic Infantile Neurological, Cutaneous and Articular (CINCA) / Muckle Wells syndrome (*n* = 1). One study involved children with an infectious cause for their uveitis (Toxoplasmosis chorioretinitis). Where uveitis site was specified (33/57 studies, 58%), the most common site of inflammation was anterior disease with or without intermediate involvement (27/57, 47%). Twenty-four of the 57 studies (42%) included children with uveitis of any anatomical category. Inclusion age ranged from 0 to 18 years. Almost 50% (29/57) of the studies excluded children aged under 6 years old (Fig. 2), but all but one study involving children with JIA included this age group.

**Fig. 3:**
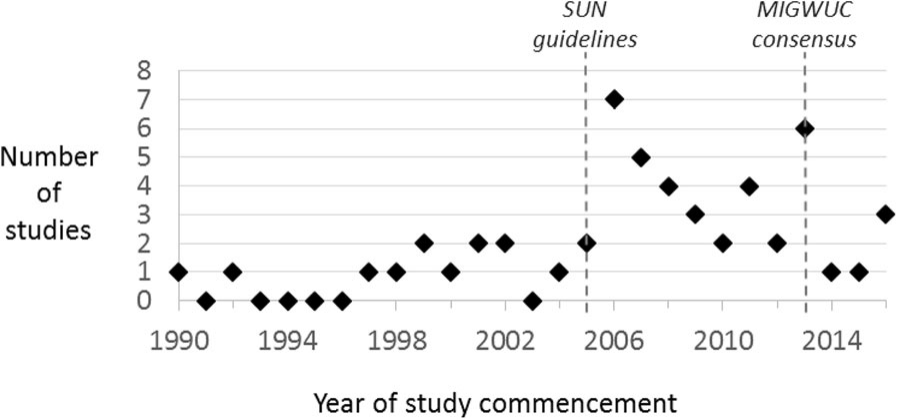
Year of study commencement for included studies. With vertical reference lines indicating publication dates of Standardised Uveitis Nomenclature (SUN) [11] guidelines, and the Multinational Interdisciplinary Working Group for Uveitis in Childhood (MIGWUC) [12] proposed outcome measures

Date of study commencement ranged from 1990 to 2016. During the 10 years prior to 2005, the publication data of the SUN consensus guidelines on uveitis classification, there were 10 new eligible studies. During the 10 years following 2005, 35 new studies started, a statistically significant increased event rate (increased rate ratio 3.5, 95% CI 1.7–7.9, *p* < 0.001, Fig. 3). There was no significant increase in study commencement rate following publication of the Multinational Interdisciplinary Working Group for Uveitis in Childhood’s Proposed Outcome Measures for Prospective Clinical Trials in 2012 [12].

### Outcome measure structure and type

In 15 of the studies, primary outcomes measures were not related to uveitis outcomes (disease activity, ocular complications, visual function, or vision related quality of life). For the remaining 42 studies, all primary outcomes involved measures of therapeutic efficacy, with a third (16/42) using a single efficacy variable to assess the impact of the intervention on children with uveitis. The structure most commonly used was multiple separate outcome measures (Table 2). In a quarter of studies, outcomes were assessed less than 3 months following the intervention. Excluding studies of post-operative uveitis, for which the median time to outcome was 3 weeks, the median time to outcome measure was 1 year (interquartile range 0.2–2 years, total range 0.01–8 years).

We identified the use of 20 different domains across 5 dimensions to determine outcome (Table 3). Disease activity was the most commonly assessed dimension (Table 3), used to assess outcome in 50 of the 57 studies. The domains examined for anterior uveitis were cell count and flare; for intermediate uveitis were vitreous cell count and haze; and for posterior uveitis were new onset of lesions, and changes on fluorescein dye angiography of posterior ocular circulation. For all sites of uveitis, activity was also assessed using the presence of macular oedema and the use of oral or topical corticosteroids.

**Table 3:** Distribution of trial registrations

Registry	Uveitis^a^	Childhood uveitis^a^	JIA^a^
National registries			
Australian New Zealand Clinical Trials Registry (ANZCTR) *http://www.anzctr.org.au/*	20	2	18
Brazilian Clinical Trials Registry (ReBec) *http://www.ensaiosclinicos.gov.br/*	0	0	1
Chinese Clinical Trial Registry (ChiCTR) *http://www.chictr.org.cn/enIndex.aspx*	5	2	0
Clinical Research Information Service (CRiS), Republic of Korea *http://cris.nih.go.kr/cris/en/use_guide/cris_introduce.jsp*	0	0	0
Clinical Trials Registry - India (CTRI) *http://ctri.nic.in/Clinicaltrials/login.php*	15	-^b^	2
Cuban Public Registry of Clinical Trials (RPCEC) *http://registroclinico.sld.cu/en/home*	4	0	1
German Clinical Trials Register (DRKS) *https://www.drks.de/drks_web/*	19	-^b^	54
Iranian Registry of Clinical Trials (IRCT) *http://www.irct.ir/*	33	1	0
Japan Primary Registries Network (JPRN) *https://rctportal.niph.go.jp/en/link*	91	6	16
Thai Clinical Trials Registry (TCTR) *http://www.clinicaltrials.in.th/*	2	0	0
The Netherlands National Trial Register (NTR) *http://www.trialregister.nl/trialreg/index.asp*	4	0	5
Sri Lanka Clinical Trials Registry (SLCTR) *https://slctr.lk/trials*	0	0	0
U.S. National Institutes of Health: USA only *https://clinicaltrials.gov/*	177	63	82
International registries			
U.S. National Institutes of Health: International *https://clinicaltrials.gov/*	328	107	192
ISCTRP *http://apps.who.int/trialsearch*	302	59	217
Pan African Clinical Trial Registry (PACTR) *http://www.pactr.org/*	0	0	0
EU Clinical Trials Register (EU-CTR) *https://www.clinicaltrialsregister.eu/*	78	9	61
Total individual studies (excluding duplications)	271	249	649

^a^Figures include studies registered across multiple registries

^b^Registry search programme did not enable search filtered participant age

### Outcome measure quality

We identified 30 studies (60%) which used reproducible methodologies to assess disease activity. The SUN grading schemes were used in 11/27 studies undertaken after 2006 where the intervention was specifically for anterior disease with or without intermediate involvement. Objective assessment of the anterior chamber was used in 2 studies (both of which used a laser flare photometry machine [23] to assess light scatter caused by the presence of inflammatory cells). Only 2/12 (18%) studies involving participants with intermediate or posterior disease used SUN grading schemes. The most commonly used outcome was vitreous cell count, (Table 4) which is not part of the SUN grading scheme [11]. Macular oedema was used as a measure of activity in 21/57 (37%) studies, with objective assessment (through optical coherence tomography retinal imaging) in 19 studies. Disease activity was assessed using a domain defined by the concurrent use of systemic or topical corticosteroid in eight studies (14%).

**Table 4:** Characteristics of outcome measures in included trials by uveitis type

	Anterior (*n* = 20)	Anterior & intermediate (*n* = 7)	Posterior (*n* = 5)	Any site (*n* = 24)	Total (*n* = 57)
Uveitis related primary outcome	18	4	3	17	42
*Safety only*	*0*	*0*	*0*	*0*	*0*
*Single efficacy*	*9*	*2*	*1*	*4*	*16*
*Multiple separate efficacy*	*7*	*2*	*2*	*11*	*22*
*Composite efficacy*	*2*	*0*	*0*	*2*	*4*
Time to outcome measure in years (IQR)	0.04–0.5	0.4–3	1–2.5	0.5–2	0.2–2
Activity	20	8	5	18	47
*ACC inactive SUN grade*	*7*	*3*	*–*	*4*	*–*
*ACC 2 step change SUN grade*	*1*	*0*	*–*	*3*	*–*
*Objective assessment AC Flare*	*1*	*1*	*0*	*0*	*–*
*Other AC assessment*	*11*	*4*	*2*	*1*	*–*
*Vitreous CC zero*	*–*	*3*	*0*	*1*	*–*
*Vitreous 2 step change SUN grade*	*–*	*0*	*0*	*2*	*–*
*Vitreous CC reduction*	*–*	*0*	*2*	*3*	*–*
*Objective assessment vitreous*	*–*	*0*	*0*	*0*	*–*
*Other vitreous assessment*	*–*	*5*	*1*	*1*	*–*
*Macular oedema (MO)*	*3*	*2*	*3*	*13*	*21*
*Objective assessment MO*	*2*	*2*	*3*	*12*	*19*
*Use of topical steroids*	*1*	*1*	*1*	*4*	*7*
*Use of systemic steroids*	*0*	*2*	*1*	*4*	*7*
Visual acuity	4	1	3	14	21
*Age appropriate measure*	*0*	*1*	*1*	*6*	*8*
Reproducible assessment of cataract	0	0	0	0	0
Reproducible assessment of glaucoma	0	0	0	0	0
Reproducible assessment of band keratopathy	0	0	0	0	0
Vision related function	1	0	0	2	3
Vision related quality of life	1	0	2	0	3
Non-specific quality of life measure	1	0	1	1	3
*Validated for use in children*	*0*	*0*	*0*	*0*	*0*

*ACC* anterior cells count, *SUN* Standardised Uveitis Nomenclature, *AC*, anterior chamber, *CC* Cell count

On subgroup analysis of the use of any reproducible assessments of disease activity before and after publication of the 2005 SUN guidance and 2012 MIGWUC guidance, although there was a higher proportion of studies using reproducible scales following publication of SUN guidance (23/35, 66%, versus 3/9, 33%) this difference was not significant.

Visual function, which for all relevant studies was measured using acuity, was utilised as a primary outcome measure for almost all studies involving posterior uveitis, but under a third of those involving only participants with anterior uveitis. In the 18 studies involving children aged under 6 years old which used visual function outcome measures, only 8/18 (44%) described the use of age-appropriate acuity assessment measures.

The incidence of structural or functional sequelae of inflammation were used as outcome measures in 12 studies. In 3 studies the incidence of cataract was specified as an outcome measure, and 4 studies used the development of glaucoma as a specific outcome event. No study described a reproducible method of assessing the presence of structural complications, such as optic disc imaging for glaucoma.

In all three studies in which vision related function measures were used to measure disease outcomes, investigators used the NEI VFQ-25 tool, which was developed for use in adults [24]. None of the five studies which assessed vision related quality of life used metrics which were validated for use in childhood, such as the Children and Young Person Vision Related Quality of Life instrument [25].

### Outcome measure characteristics by study completion and dissemination status

**Fig. 4:**
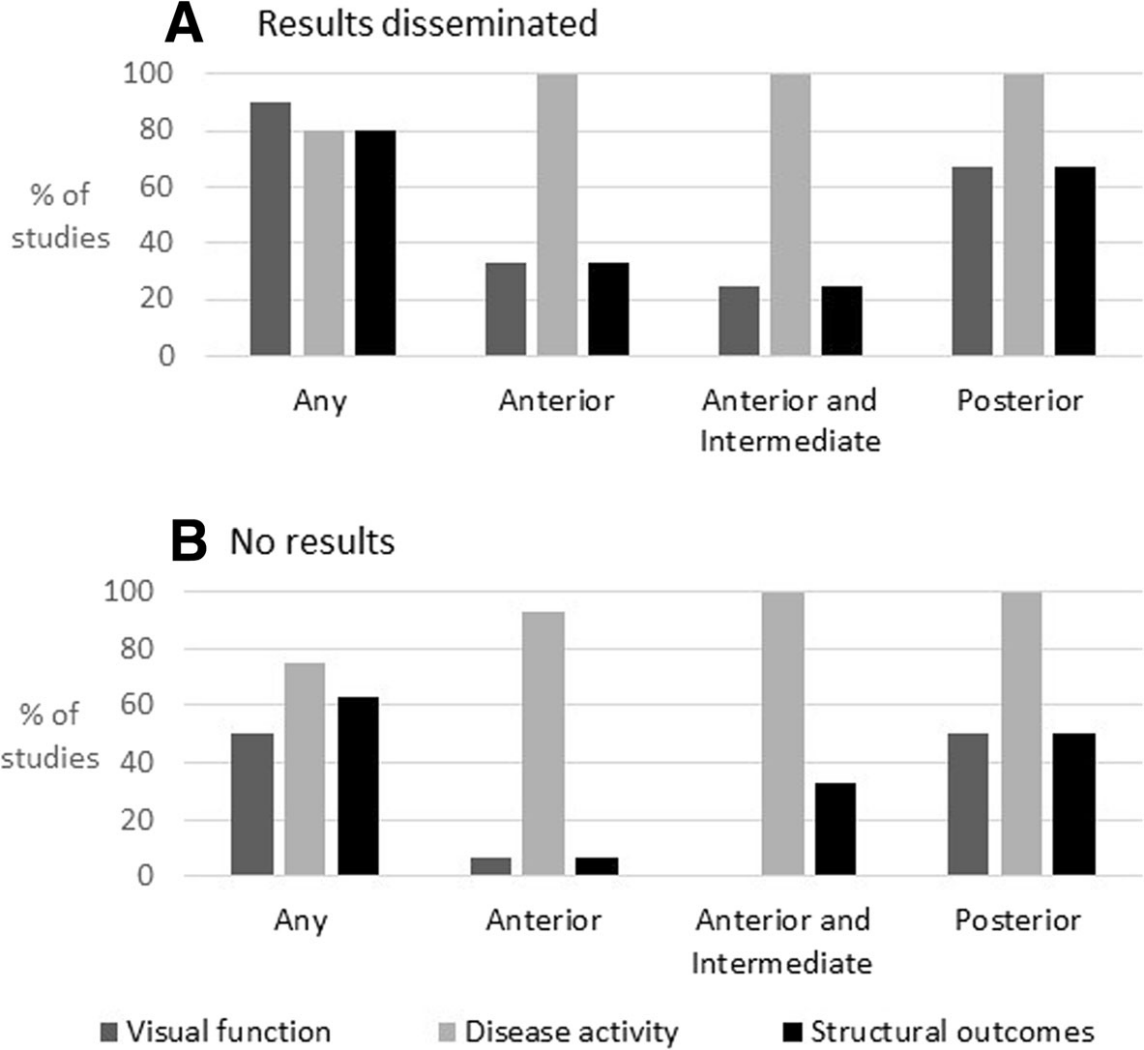
Proportion of studies assessing the different outcome dimensions, by inflammation site and by study dissemination status

Of the 57 studies, 27 were described as completed and had published their findings either directly within their registry entry (*n* = 3) or through a publication linked to the entry (*n* = 11). Contrary to ICMJE guidance, 13/27 studies had disseminated their findings through publications which were not linked to their registry entry, and which were only identified through the accompanying review of published randomised controlled trials. Status was described as completed but without available results for 8 studies, active or ongoing for 14 studies, and unknown for 7 studies. One study had been terminated due to low enrolment rates. Dimensions used in studies which had published results were compared to those which had not yet disseminated outcomes (Fig. 4). In order to provide an appropriate comparison by only including studies which had sufficient time post study completion in order to disseminate findings, we excluded studies with an estimated completion date of later than January 2015. Although, across all uveitis types, all of the three most commonly assessed dimensions (visual acuity, disease activity, structural sequelae of inflammation) were all more likely to be utilised in studies which had disseminated results, our study sample size gives this review insufficient power to determine whether this difference is statistically significant.

## Discussion

This review of outcome measures used in interventional trials involving children with or at risk of uveitis has identified considerable heterogeneity in metric type, structure, and time at which outcome was assessed. This heterogeneity may be a reflection of the poor quality of the outcome measures used: 40% of studies used non-reproducible methodologies to assess disease activity outcomes, and the outcome measures which related to dimensions such as quality of life, structural complications, or functional sequelae of uveitis, were not validated for use in childhood.

Our review searched within registries of interventional trials, rather than within the published literature, in order to overcome the obstacle of publication bias and the outcome reporting bias, in which ‘negative’ trials, and ‘negative’ outcomes are not reported respectively [26]. Consequently, studies which have not been registered on an ICMJE recognised system have not been evaluated. As registration has, since 2005, been a pre-requisite for publication within an ICJME journal, it is unlikely that our approach has resulted in significant omissions of ongoing or completed studies. Full details of study methodology were not always available through the ICMJE-recognised registries. Although we sought further details through a supplementary search of the published literature and attempted communications with registered principal investigators for methodology details, we may be missing details which clarify the reproducibility of the metrics chosen by the reported studies.

The annual registration rate of trials involving children with uveitis increased significantly after 2005, that is in the years following the publication of the SUN guidelines. There are a number of possible explanations for this: first it may be that the registration of a number of trials in planning phase were deferred until the widely-anticipated SUN consensus had reported; second, it may be that the SUN consensus itself stimulated a number of trials by providing a much needed structure, reproducibility, and specificity to the measurement of disease activity; thirdly, this could be a chance finding. We also recognise that studies which commenced prior to 2005 and which were not registered within the ICMJE may have been omitted from our review.

Rare disease interventional research faces the challenge of adequate study recruitment to ensure sufficient sample sizes, exacerbated by the measurement and selection biases at play in the study of complex disease states. Additionally, small numbers and other methodological and logistical challenges constrain researchers’ ability to undertake randomised controlled trials. Reproducible, precise and validated outcome metrics are key to study feasibility, as are surrogate endpoints. Surrogate endpoints are a particular concern in paediatric rare disease research: the developmental trajectory of children with rare disease is often disturbed, weakening our ability to prognosticate on findings reported early in the disease course. Other obstacles to paediatric rare disease research include the appropriateness of outcome measures at different developmental stages. Almost 50% of all the studies excluded children aged under 6 years old, which may be a reflection of the difficulty in capturing outcome for young children. However, younger children are at particular risk of disease: JIA typically presents in children aged under 6 years, and this multisystem disease has a uveitis prevalence of 20–30% [7, 27, 28], with young age at presentation being a risk factor for poor outcomes in JIA associated uveitis [4, 6, 28]. Despite uveitis being a frequent extra-articular manifestation of JIA, we identified 184 interventional trials of children with JIA which did not include uveitis as an outcome measure, suggesting an importance evidence gap for research on this population. This disconnect between JIA and childhood uveitis research may be another obstacle to the translation of research into clinical care.

The absence of robust evidence on the long term clinical significance of different levels of inflammation in uveitis, and the imprecision and insensitivity of the current metrics of disease activity, complicate the use of inflammation as a surrogate endpoint for visual disability. The long duration of follow up necessary to capture uveitic visual loss, and the irreversibility of uveitic visual loss, make visual function a challenging end point for interventional childhood inflammatory disease research, and highlight the importance of developing a validated outcome able to predict final visual function (a ‘surrogate endpoint’). For children with uveitis, the majority of whom (80%) [1, 7] have chronic anterior disease, disease activity is the most commonly measured outcome dimension in interventional clinical research, and is used as a surrogate endpoint for visual disability. There is evidence that chronic disease activity results in visual disability, but the dose-response relationship is unclear, particularly for those with milder degrees of inflammation. There is also absence of clarity on the key end point for disease activity: ie whether inactive disease, or significant reduction in disease activity is the more appropriate measure of effectiveness [4, 6]. Additionally, the grading scales currently used to quantify activity are subjective, semi-quantitative, non-linear, open to intra- and interobserver variability [29], and have not been validated for use in children [7]. Objective disease activity metrics would support research on the prognostic impact of disease activity, as well as providing a precise, accurate and reproducible outcome measure. Laser flare photometry, in which the scatter of light by products within the eye is measured by a desktop instrument, has been successfully used to quantify disease activity [23, 30], and in recent trials were used to assess the efficacy of adalimumab in children with chronic anterior JIA associated uveitis [13, 31]. Laser flare meters provide an objective machine-based metric of disease activity, but have had little adoption into routine clinical practice due to expense, perceived difficulty of use, limited use beyond anterior chamber assessment, and the inability of these instruments to quantify anterior chamber cell counts. Optical coherence tomography (OCT) photography, a non-contact near infra-red high resolution imaging system, which is able to detect blood cells and proteinaceous exudate within the intraocular spaces, may have a future role in the quantification of disease activity in uveitis [32–37].

In order to impact on clinical practice, interventional trials must evaluate outcomes which are meaningful to patients as well as practitioners. There has been little work on the importance of various outcome measures to children and families with uveitis, and a paucity of validated patient reported outcome measures (PROMs) for this group. Such work is necessary if clinical trials are to translate into improvements for affected children. There is also absence of clarity within the paediatric uveitis clinical research community on PROMs, with functional assessments (which measure the impact of disease on the child’s ability across different dimensions) conflated for quality of life metrics (which enable the child to quantify the self-perceived negative impact of their disease on their life) [12].

Multiplicity of outcome measures and use of composite outcomes can dilute the ability of individual RCT findings to support clinical care, and outcome metric heterogeneity is an obstacle to the synthesis or meta-analysis of the existing literature. Inconsistency of outcome selection also leads to clinical trials with unnecessarily large sample sizes, and to reporting biases [19, 38]. The Core Outcome Measures in Effectiveness Trials (COMET) initiative, which aims to collate and stimulate resources for the development and application of core outcome sets for clinical research, has gained interest and prominence since its establishment in 2011, with sustained growth in use of COMET resources [39]. Of the 1033 references relating to planned, ongoing and completed work on determining ‘Core Outcome Sets’ currently collated on the COMET initiative website, 18 refer to eye or vision disorders [40].

In summary, our review reports on the paucity of reproducible, age appropriate and patient reported outcome measures in childhood uveitis interventional research, and significant heterogeneity of utilised outcome measures. Although the clinical research community is working on COMET supported consensus based and patient centred approaches to outcome measures in adult uveitis [41], these advances may not translate to affected children. Clinicians and researchers interested in improving outcomes for children with uveitis must identify a patient and family centred core outcome set. This will need the active involvement of children and families in priority setting, and the outcome domains will need to consider the child’s developmental stage, and the duration of follow up at which the outcome is measured. Work will then be needed to validate these objective and / or patient reported outcome measures.

## Additional file

Additional file 1: Database details and search methodology. (DOCX 16 kb)

## Data Availability

The datasets used and/or analysed during the current study are available from the corresponding author on reasonable request.

## Abbreviations

AC: Anterior chamber
ACC: Anterior (chamber) cell count
ICMJE: International Committee of Medical Journal Editors
JIA: Juvenile idiopathic arthritis
MIGWUC: Multinational Interdisciplinary Working Group for Uveitis in Childhood
RCT: Randomised controlled trial
SUN: Standardised Uveitis Nomenclature
