# Supplementary material for: Under-utilisation of reproducible, child appropriate or patient reported outcome measures in childhood uveitis interventional research
Source: Orphanet J Rare Dis. 2019 Jun 6;14:125. doi: 10.1186/s13023-019-1108-3 (PMC6555041; doi:10.1186/s13023-019-1108-3)
Supplement: Supplementary file 1 — Database details and search methodology. (DOCX 16 kb) [file 13023_2019_1108_MOESM1_ESM.docx]

Additional file 1: Database details and search methodology

1. International Committee of Medical Journal Editors (ICMJE) database approval criteria (available at www.who.int/ictrp/network/trds/en/index.html )

The ICMJE states that it endorses those registries that meet the following requirements:

- accessible to the public at no charge
- open to all prospective registrants
- managed by a not-for-profit organization
- have a mechanism to ensure the validity of the registration data
- are electronically searchable
- and include a minimum 20-item trial registration dataset at the time of registration and before enrollment of the first participant

1. Trial registration search methodology

International databases approved by the International Committee of Medical Journal Editors:

| Australian New Zealand Clinical Trials Registry (ANZCTR) | http://www.anzctr.org.au/ |
| --- | --- |
| Brazilian Clinical Trials Registry (ReBec) | http://www.ensaiosclinicos.gov.br/ |
| Chinese Clinical Trial Registry (ChiCTR) | http://www.chictr.org.cn/enIndex.aspx |
| Clinical Research Information Service (CRiS), Republic of Korea | http://cris.nih.go.kr/cris/en/use_guide/cris_introduce.jsp |
| Clinical Trials Registry - India (CTRI) | http://ctri.nic.in/Clinicaltrials/login.php |
| Cuban Public Registry of Clinical Trials(RPCEC) | http://registroclinico.sld.cu/en/home |
| EU Clinical Trials Register (EU-CTR) | https://www.clinicaltrialsregister.eu/ |
| German Clinical Trials Register (DRKS) | https://drks-neu.uniklinik-freiburg.de/drks_web/navigate.do?navigationId=search&reset=true |
| Iranian Registry of Clinical Trials (IRCT) | http://www.irct.ir/ |
| Japan Primary Registries Network (JPRN) | http://rctportal.niph.go.jp/en/ |
| Thai Clinical Trials Registry (TCTR) | http://www.clinicaltrials.in.th/ |
| The Netherlands National Trial Register (NTR) | http://www.trialregister.nl/trialreg/index.asp |
| Pan African Clinical Trial Registry (PACTR) | http://www.pactr.org/ |
| Sri Lanka Clinical Trials Registry (SLCTR) | http://www.slctr.lk/ |
| U.S. National Institutes of Health: USA only | https://clinicaltrials.gov/ |
| U.S. National Institutes of Health: International | https://clinicaltrials.gov/ |
| ISCTRP | http://apps.who.int/trialsearch |

Search date: 10^th^ March 2017

1. Searched using the keyword / term

‘Uveitis’

1. Searched using keyword / term

‘Juvenile Idiopathic Arthritis’

Total studies identified n=564

1. Supporting searches of published literature

Ovid MEDLINE In-Process & Other Non-Indexed Citations and Ovid Medline

Search date: 10/6/17, n=137

1. (randomi?ed or randomi?ed control* trial*).tw.
2. Randomized Controlled Trials as Topic/
3. Clinical Trial as Topic/
4. 1 or 2 or 3
5. exp Child/
6. exp Adolescent/
7. exp Young Adult/
8. Adolescen*.tw.
9. Young person*.tw.
10. Boy*.tw.
11. Girl*.tw.
12. teen*.tw.
13. Schoolchild*.tw.
14. Young adult*.tw.
15. Youth*.tw.
16. P*ediatric*.tw
17. Student*.tw.
18. Pupil*.tw.
19. Juvenile*.tw.
20. 5 or 6 or 7 or 8 or 9 or 10 or 11 or 12 or 13 or 14 or 15 or 16 or 17 or 18 or 19
21. Uveitis.tw.
22. 4 and 20 and 21

Cochrane Central Register of Controlled Studies (CENTRAL; 2016, Issue 5) in the Cochrane Library

Search date: 10/6/17, n=67

1. Uveitis:ti,ab
2. Child*:ti,ab
3. Adolescen*:ti,ab
4. Juvenile*:ti,ab
5. Young person*:ti,ab
6. #2 or #3 or #4 or #5
7. #1 and #6
